# A Fetus with Congenital Microcephaly, Microphthalmia and Cataract Was Detected with Biallelic Variants in the *OCLN* Gene: A Case Report

**DOI:** 10.3390/diagnostics11091576

**Published:** 2021-08-30

**Authors:** Vivian Kwun Sin Ng, Tze Kin Lau, Anita Sik Yau Kan, Brian Hon Yin Chung, Ho Ming Luk, Wai Fu Ng, Mengmeng Shi, Kwong Wai Choy, Ye Cao, Wing Cheong Leung

**Affiliations:** 1 Department of Obstetrics and Gynaecology, Kwong Wah Hospital, Hong Kong, China; vivian_nks@hotmail.com; 2 Department of Obstetrics and Gynaecology, Prince of Wales Hospital, The Chinese University of Hong Kong, Hong Kong, China; tklau2019@gmail.com (T.K.L.); shimengmeng@link.cuhk.edu.hk (M.S.); richardchoy@cuhk.edu.hk (K.W.C.); 3 Department of Obstetrics and Gynaecology, Queen Mary Hospital, Hong Kong, China; kansya@hku.hk; 4 Department of Paediatrics and Adolescent Medicine, The University of Hong Kong, Hong Kong, China; bhychung@hku.hk; 5 Clinical Genetic Service, Department of Health, Hong Kong, China; luksite@gmail.com; 6 Department of Anatomical and Cellular Pathology, Hong Kong Children Hospital, Hong Kong, China; ngwaifu@ha.org.hk; 7 Department of Paediatrics, The Chinese University of Hong Kong, Hong Kong, China

**Keywords:** *OCLN* gene, whole genome sequencing, microphthalmia, microcephaly, cataract, prenatal diagnosis

## Abstract

Microcephaly and microphthalmia are both rare congenital abnormalities, while concurrently, these two are even rarer. The underlying etiology would be complex interplaying between heterogeneous genetic background and the environmental pathogens, particularly during critical periods of early tissue development. Here, we reported a prenatal case with microcephaly, microphthalmia, and bilateral cataracts detected by ultrasonography and confirmed by autopsy. Various routine infection-related tests and invasive genetic testing were negative. Whole genome sequencing of fetus and parents revealed *OCLN* gene defects may be associated with these multiple congenital abnormalities.

## 1. Introduction

Congenital microcephaly is defined as occipitofrontal head circumference (OFC) more than two standard deviations (SD) below the mean adjusted for age, sex, and race, which includes approximately 2% of the population [1]. In severe (true) case, it is defined by OFC ≤−3 SD from the mean, which includes approximately 0.1% of the population [1]. The overall prevalence of microcephaly in the absence of Zika virus infection was 3 to 7.4 per 10,000 live births [2,3]. The etiology is diverse but can be divided into environmental (e.g., congenital infection, teratogen exposure, trauma resulting in ischaemia) and genetic causes (e.g., chromosomal abnormalities leading to cortical malformation, single gene mutation leading to neuronal migration disorders and syndromes) [4].

Microphthalmia is defined by the reduced total axial length of the globe within the orbit. It may be unilateral or bilateral. Together with anophthalmia (absence of ocular tis-sue within orbit), the prevalence of congenital microphthalmia is approximately one to three per 10,000 live births, according to several countries’ or regions’ population-based assessments [5,6,7]. It accounts up to 11% of blind children [8]. It may occur in isolation or as part of a syndrome in more than 50% (published range 33–95%) of cases that present extraocular findings of variable severity, most commonly involving craniofacial region with anomalies, musculoskeletal system [9]. The etiology of microphthalmia is complex, as it may be caused by genetic abnormalities (e.g., gene mutation, chromosomal aberration), infection, or exposure of teratogens. Several risk factors, including advanced maternal age, elevated maternal pre-pregnancy body mass index (BMI), maternal smoking during pregnancy, multiple births, preterm birth, and low birth weight have been suggested [7].

Prenatal detection of microcephaly and microphthalmia is challenging but possible with the use of high-resolution ultrasound, magnetic resonance imaging [10,11]. The presence of orbit shown in imaging does not guarantee normal visual function, as it depends not only on orbit but also on retinal development and other intra- or extra-ocular characteristics. Moreover, the neurological outcome of prenatal detected microcephaly cannot be ascertained since fetal brain is still under development. Microcephaly or microphthalmia could arise independently or together as presentations of certain rare syndromes. Therefore, establishing molecular diagnosis toward an informative prenatal counselling regarding multiple congenital abnormalities is important but difficult considering their rarity and heterogeneous etiologies.

The formation of brain and orbit involves multiple processes of induction and differentiation during embryogenesis, which involved hundreds of genes [12,13]. Its genetic heterogeneity and rarity make diagnosis challenging and usually requires a comprehensive genome-wide genetic testing which stand a better chance to pinpoint the diagnosis. Here, we present an undiagnosed case of fetal bilateral microphthalmia, in which microcephaly carries biallelic variants in the *OCLN* gene identified by whole genome sequencing.

## 2. Case Report

**Figure 1:**
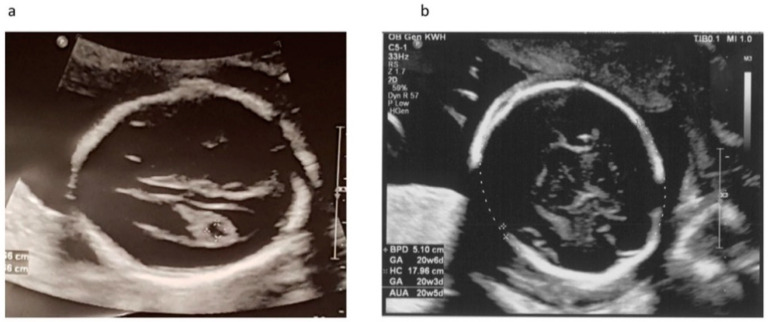
Ultrasound scan at 21 and 23 weeks of gestation. (**a**) Transventricular plane at 21 weeks showed unilateral choroid plexus cyst which was resolved later in scan at 23 weeks; (**b**) Biparietal diameter and head circumference at 23 weeks.

**Figure 2:**
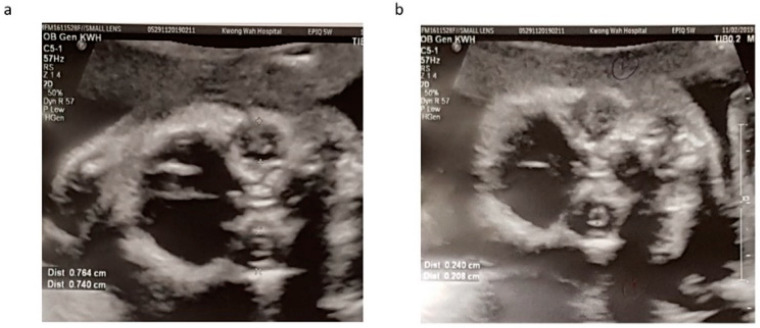
Orbital (**a**) and lens (**b**) diameters measured at 21 weeks with reference to growth chart by Goldstein et al. Both orbital and lens diameters were much smaller than 10th centile.

**Figure 3:**
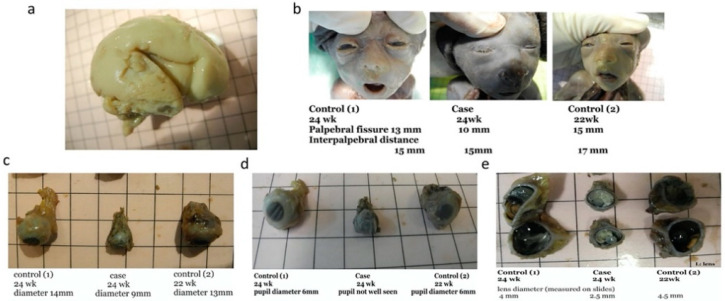
Postmortem examination of abortus confirmed ultrasound diagnosis of microcephaly and microphthalmia. (**a**) Gross feature of the abortus brain shows no evidence of other anomalies, disruption or evidence of infection to support syndromic conditions or congenital infection; (**b**) Palpebral fissure of abortus is shorter compared to control 1 (**left**) and 2 (**right**) at similar gestation; (**c**) Lateral view of the eyeball of abortus with comparison to controls at 22 weeks (**right**) and 24 weeks (**left**); (**d**) Pupil of abortus comparing with controls at 22 weeks (**right**) and 24 weeks (**left**). The pupils were not well seen in abortus; (**e**) Dissected eyeball demonstrating the small and opacity lens of our abortus with comparison to controls at 22 weeks (**right**) and 24 weeks (**left**).

Our patient was a 37 year-old G2P1 Chinese female. Her first pregnancy was a pre-term delivery at 35 weeks of gestation of an otherwise healthy girl. During this pregnancy, she suffered from roseola and was exposed to Erythromycin at very early gestational age. At first trimester, non-invasive prenatal testing (NIPT) for common aneuploidies and mi-cro-deletion/duplications showed low-risk results. The fetal morphology scan at 21 weeks of gestation showed a unilateral choroid plexus cyst in the transventricular plane (Figure 1a), which was resolved later. The diameters of both orbits and lens (Figure 2a,b) were smaller than 10th centile using the nomogram by Goldstein et al. [14]. Invasive genetic testing was suggested, and the patient agreed to amniocentesis. Quantitative fluorescence polymerase chain reaction (QF-PCR), karyotyping, and chromosomal microarray analysis (CMA) all indicated a chromosomally normal male fetus. Screening for congenital infection (toxoplasmosis, rubella, cytomegalovirus (CMV), herpes simplex virus (HSV), human immunodeficiency virus) from maternal blood and urine as well as amniotic fluid was negative at the time of diagnosis. In the later scan at 23 weeks of gestation, the fetal head circumference (Figure 1b) was found three weeks smaller than expected. The patient decided to terminate the pregnancy at 23 weeks of gestation after counselling. The fetal postmortem examination (Figure 3a–e) confirmed microcephaly in the absence of brain anatomy disruption, calcification, and fetal akinesia. Bilateral microphthalmia with poorly formed anterior chamber and lens closely opposed to the corneal surface. The corneas and lens were opacified suggestive of bilateral cataract. There was no evidence of infection in any organs.

**Figure 4:**
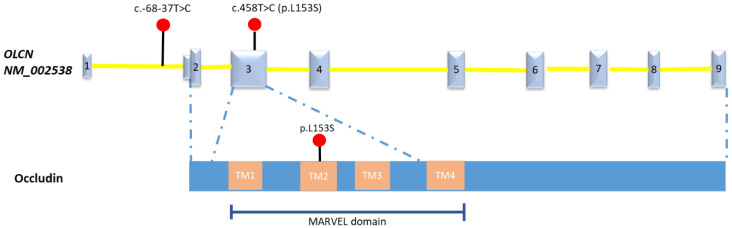
The schematic of mutations in OCLN gene, Occludin detected in this fetus. Number 1-9 indicate the Exon 1-9 of the OLCN gene (NM_002538). TM: transmembrane domain.

In the absence of chromosomal abnormalities identified by NIPT, QF-PCR, and karyotyping and the lack of pathogenic copy number variant detected by CMA, trio whole exome sequencing was performed but did not yield relevant alternations. In order to further explore the possibility of an underlying genetic cause, the couple was counselled and agreed to whole genome sequencing (WGS, 30×) in a research setting. The analysis of single nucleotide variants (SNVs), small indels, copy number variants (CNVs), and structural variants (SVs) were conducted by our published in-house pipelines [15]. The clinical significance of the detected variants was interpreted in accordance with the guidelines of the American College of Medical Genetics and Genomics (ACMG) [16,17]. This trio WGS data analysis did not detect any clinically significant chromosomal balanced rearrangement or loss of heterozygosity (>5 Mb) but revealed rare compound heterozygous variants in the *OCLN* gene (NM_002538) that might be related to the fetal abnormalities: maternally inherited c.458T>C (p.L153S) and paternal inherited c.-68-37T>C (Figure 4). Defects in *OCLN* cause Pseudo-TORCH syndrome 1 [MIM:251290], an autosomal recessive neurologic disorder with characteristic features including congenital microcephaly, intracranial calcifications, and severe developmental delay, which mimic intrauterine TORCH infection in the absence of evidence for infection [18]. The *OCLN* gene c.458T>C (p.L153S) variant was extremely rare with minor allele frequency 5.5 × 10^−4^ in the East Asian ethnic population according to the gnomAD. Multiple in silico algorithm predicted this missense having deleterious impact on protein (Polyphen: probably_damaging; SIFT: deleterious, REVEL: 0.51; CADD: 25.5). This variant is highly conservative throughout different species with phyloP100wayAll score up to 7.887. The c.-68-37T>C variant was located in the intron 1 with a CADD score of 8.1. This variant has never been reported before. Both variants were classified as variants of uncertain significance (VUS) according to the ACMG guidelines (criteria PM2 was applied for both variants) [16].

On the other hand, this patient was pregnant soon after the termination and was referred for genetic counselling by clinical geneticist. The couple was informed of the WGS findings that variants of uncertain significance in the *OCLN* gene were identified. This result suggested that these variants could not explain clearly the fetal abnormalities. It would be due to the gaps in our current knowledge that impede an accurate interpretation of their pathogenicity. Prenatal invasive diagnosis for the compound heterozygous variants was not indicated if sonographic evidence did not show microcephaly, microphthalmia, or other abnormalities. The couple understood the genetic results and the limitation of current testing. They preferred a conservative approach with ultrasound monitoring after genetic counselling. Her pregnancy went on uneventfully with serial ultrasounds showing normal growth of both fetal orbits and lens. She had delivery at term by lower segment caesarean section in the private sector. There was no abnormality found in the newborn.

## 3. Discussion

Here, we presented a prenatal case with microcephaly, microphthalmia, and bilateral cataracts detected with rare biallelic changes in the *OCLN* gene through whole genome sequencing analysis. The *OCLN* gene identified encodes an integral membrane protein at tight junctions (TJs), Occludin, which is functional in endothelium in early fetal development and maintenance of blood–brain barrier in postnatal life [18,19]. Recently, Bendriem et al. revealed that *OCLN* regulated the centrosome organization and dynamics which is required by the early corticogenesis [19]. Multiple genes related with centrosome associated functions are known to cause microcephaly disorders [18]. The complex role of Occludin has been demonstrated in mice models with occludin deficiency that heterogeneous phenotypic effects impact gastric epithelium, brain, testes, salivary gland, as well as compact bone [19]. Occludin deficiency due to *OCLN* gene mutation leads to abnormal cerebral vasculature and blood–brain barrier, which results in immune cell mediated insults and ischaemia and thus cortical malformation. The variant c.458T>C (p.L153S) was predicted as disease-causing by multiple in silico algorithms. It was located in the highly conserved MARVEL domain of Occludin which was commonly detected with mutations in patients [20]. Currently limited cases with biallelic changes in the *OCLN* genes were reported [21,22,23]. The patients mainly present with microcephaly, simplified gyration and polymicrogyria (PMG), intracranial calcifications, early onset seizures, and severe developmental delay which mimic congenital TORCH infection [21]. Some patients present a characteristic prominent band of cortical gray matter calcification in the brain MRI [21]. Besides the core phenotypes, other uncommon phenotypes include congenital cataract and/or microphthalmia, central diabetes insipidus, and renal dysfunction [20,21,22,23]. In general, the prognosis of patients was poor, and early death was frequently reported [22]. In comparison, our case presented partial core phenotypes, including microcephaly with general normal anatomy and microphthalmia in the 21 weeks of gestational age, with no signs regarding intracranial calcification. However, there is a paucity of data regarding prenatal presentations of *OCLN*-related syndrome. Although in silico analysis is supportive, biological studies of these variants are needed to elucidate how these mutants impact the protein function and related to patient’s phenotype. These two variants in the *OCLN* gene were classified as variants of uncertain significance, which possibly expands the phenotype spectrum to fetal microcephaly, microphthalmia, and bilateral cataracts.

Microcephaly has been stratified based on the etiology and the timing of onset. Besides infections, genetic factors would be the common causes for congenital microcephaly. Diverse genomic abnormalities cause abnormal neuronal development and migration, which commonly lead to microcephaly [1,4]. In addition, Zika virus infection could also lead to congenital microcephaly. Studies found that Zika virus affects the junctional integrity of human brain microvascular endothelial cells to enter the brain, which suggested the important physiological role of the tight junction proteins in human epithelial cells [24,25]. It also correlates the potential relationship between the abnormal tight junctions and microcephaly. However, the certain TJ protein-encoding genes such as *JAM3* produce brain hemorrhage rather than microcephaly, which the *OCLN* gene may have developmental functions unanticipated for a TJ protein [19].

Microphthalmia is a rare congenital abnormality, and prenatal diagnosis of this condition is complex and tends to be identified late as the fetal orbit and lens can only be visualized sonographically in the second trimester. A normal ultrasound finding does not guarantee that the optic nerve and visual function preserves. Eye development mainly takes place between week 3 and week 10 of embryo and involves multiple progenitors, including ectoderm, neural crest cells, and mesenchyme, which differentiate into various components of the eye and orbit [26]. Microphthalmia may not always lead to poor vision, but is associated with other ocular defects such as congenital cataracts, which are due to improper growth of the lens fibers. Increasing numbers of genetic changes associated with both syndromic and isolated microphthalmia have been reported [27]. Recently, studies suggest that the disruption of signaling in neural crest cells can lead to alterations in neural epithelial derived optic cup formation, resulting in microphthalmia [28,29]. TJ proteins, especially those with MARVEL (MAL and related proteins for vesicle traffic and membrane link) domain are usually regulators or components of many important signaling pathway. MARVEL domain proteins may modulate junctional permeability properties of cells and send signals through tight junctions into the cell interior [30]. MarvelD3, which belong to the closely associated MARVEL protein family, the same as Occludin, has recently been identified to be required to attenuate JNK signaling pathway where direct JNK stimulation disrupts neural crest development and links tight junctions and modulation of the JNK pathway to eye morphogenesis [30,31]. Occludin also contributes to the compartmentalization of the ocular micro-environments by controlling the flux through the retinal–blood barrier in Xenopus [31]. These imply the potential pathophysiological roles of Occludin in human eye development.

The genome sequencing technology is migrating rapidly from research studies in the clinical application. However, accurate assessment of genetic variations to prioritize the disease-causing mutations remains challenging. Whole genome sequencing could in-crease the diagnostic yield to 40–50% of cases; this improvement is substantial compared with ~25% of exome sequencing [32,33]. Still, more than half of cases would receive uncertain or negative results, which means no explanatory genetic variant detected. A negative or uncertain result may be due to current interpretive limitations that prioritize the disease-causing variants from overwhelmingly large targets [34]. Currently, a phenotype-driven strategy is widely adapted for process of genome scale sequencing data to help select relevant disease genes on the basis of known phenotype–gene associations [35]. However, prenatal presentations of most genetic diseases were very limited, as in this case, which may hamper data interpretations of prenatal genetic findings. The functional impacts of those identified variants through experimental characterization or in silico approaches would be an important step in genome interpretation. Nevertheless, a future reanalysis could be considered to reassess the genetic causes with multilayered and more updated information [36].

In conclusion, here, we reported a prenatal case with microcephaly, microphthalmia, and bilateral cataracts, with detection of rare biallelic *OCLN* variants by whole genome sequencing. Given WGS provided a more comprehensive genome-wide investigation compared to CMA and whole exome sequencing in prenatal diagnosis, and less DNA was required for the experiment, our case report supports that WGS would be considered the second-tier genetic testing assay in prenatal diagnosis and the potential migration to early gestational week while proper pre- and post-test genetic counseling is warranted.

## Data Availability

The data presented in this study are available on request from the corresponding author. The data are not publicly available due to privacy and ethical restrictions.
